# The complete mitochondrial genome of *Glyptotendipes tokunagai* (Arthropoda; Insecta; Diptera; Chironomidae)

**DOI:** 10.1128/mra.01101-25

**Published:** 2026-02-03

**Authors:** Xinyue Huang, Dihao Huang, Hengying Chen, Chunmei Zhu, Wenhao Wang, Shan Ouyang, Xiaoping Wu, Chunhua Zhou

**Affiliations:** 1 School of Life Sciences, Nanchang University 621984, Nanchang, China; University of Maryland School of Medicine, Baltimore, Maryland, USA

**Keywords:** *Glyptotendipes tokunagai*, mitochondrial genome, China

## Abstract

We present the complete mitochondrial genome sequence of *Glyptotendipes tokunagai* in this study. This circular genome encompasses 15,241 base pairs and exhibits a high AT content of 77%. It contains a total of 13 protein-coding genes, 22 transfer RNA (tRNA) genes, and 2 ribosomal RNA (rRNA) genes.

## ANNOUNCEMENT

*Glyptotendipes tokunagai* is an aquatic insect of the family Chironomidae (Diptera), with larvae inhabiting aquatic ecosystem sediments (1). As a representative Chironomidae species, it is closely linked to freshwater quality, serving as a key bioindicator for freshwater ecosystem assessment and widely used in aquatic environmental protection research (2). This study reports its complete mitochondrial genome, providing a valuable basis for advancing systematics, population genetics, and database construction.

The sample was collected from Poyang Lake (GPS coordinates: 29.253°N, 116.190°E) in 2023 using a Peterson grab sampler (1/16 m²). Sediment samples were washed through a 40-mesh sieve, and macroinvertebrates were manually selected from a white porcelain dish. The specimens were preserved in absolute ethanol at low temperature (4°C). Genomic DNA was extracted from a single, whole specimen using the Rapid Animal Genomic DNA Isolation Kit. The library was constructed with the Hieff NGSMaxUp II DNA Library Prep Kit for Illumina. Sequencing was carried out on the Illumina NovaSeq 6000 platform, generating paired-end reads of 150 bp in length. A total of 49,016,436 raw reads were generated, corresponding to 7,352 Mbp of sequencing data with an average coverage depth of 1,371×. Quality control was conducted using fastp v0.36 (3) to remove adapter sequences, low-quality reads (where >40% of bases had a quality score < Q15), and paired-end reads shorter than 35 nt. To assess potential contamination, 10,000 randomly selected reads were aligned to the NCBI NT database using blastn, with *Chironomus tepperi* (JN861749.1) as the top-matching species (4). *De novo* assembly was carried out with SPAdes v3.15 (5), with GapFiller v1.11 (6) for gap closure and Pilon v1.24 (7) for base-error correction. Gene annotation was performed using MITOS v1.1.7 with the Invertebrate Mitochondrial Code (8). We used the homology with the reference genome to determine the most likely start and stop codons, and the determination process was clear. Manual curation refined gene boundaries through NCBIBLAST comparisons with reference mitochondrial genome of *Glyptotendipes caulicola* (NC_016167.1). The assembled genome showed 87% identity to the genome of *G. caulicola*.

**Fig 1:**
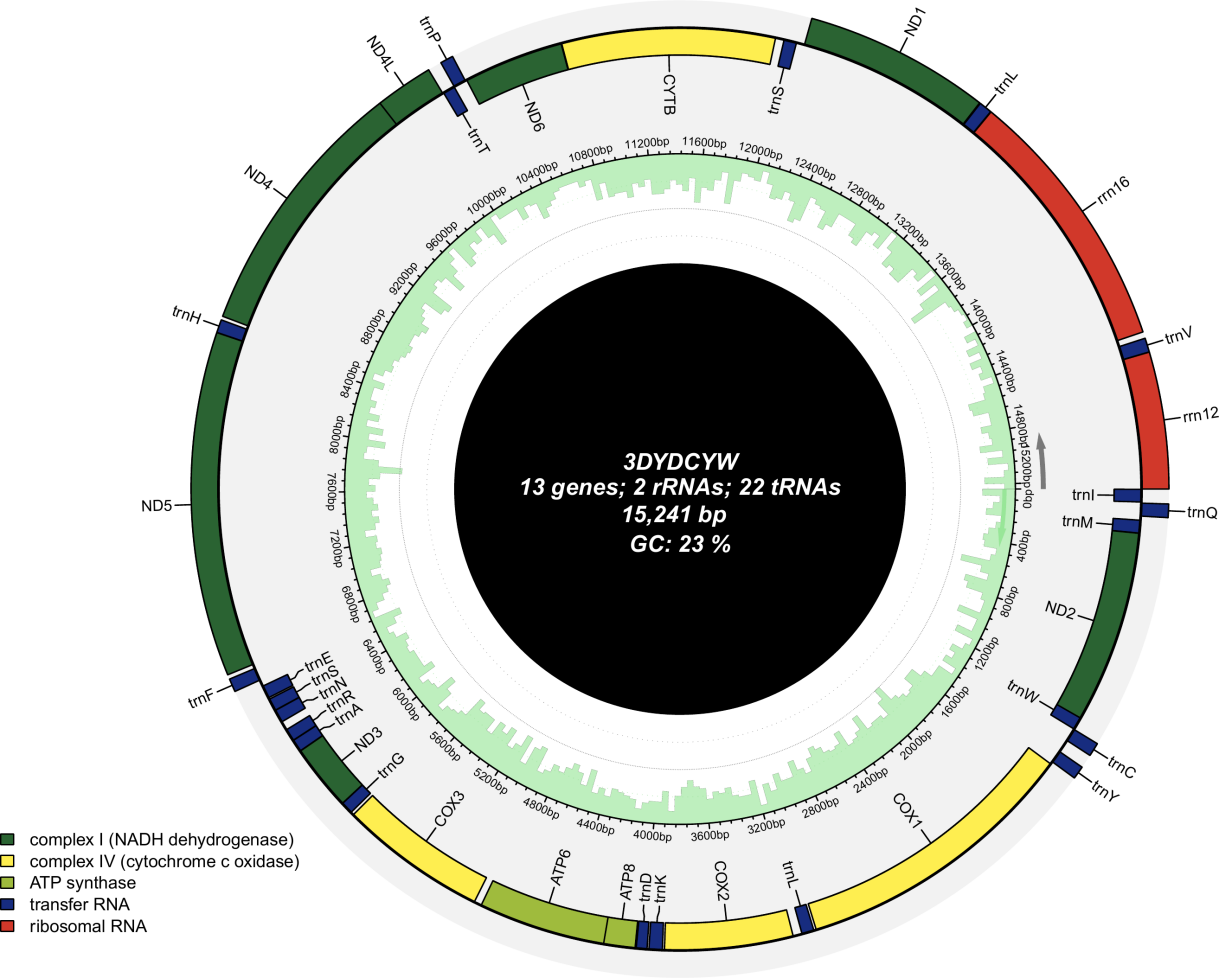
Mitochondrial genome map of *Glyptotendipes tokunagai*. The direction of transcription is indicated by arrows. The color coding corresponds to different genome groups, and the specific information is shown in the legend at the bottom left corner. The outermost ring displays the genes, and the innermost ring shows the GC content in light green. Genes transcribed clockwise are located on the inside of the circle, while genes transcribed counterclockwise are on the outside.

The complete mitochondrial genome was 15,241 bp (GenBank accession number PV781185). Protein-coding genes have 77% AT and 23% CG content, showing significant AT bias. It included 13 PCGs, 2 ribosomal RNA (rRNA) genes (12S and 16S), and 22 transfer RNA (tRNA) genes (Table 1). PCGs used diverse start codons: ATG (COX2, ATP6, COX3, ND4, ND4L, and CYTB), ATT (ND2, ATP8, ND3, ND6, and ND1), TTG (COX1), and GTG (ND5). ND5, ND4, ND4L, and ND1 were on the heavy strand; COX2, ATP6, COX3, CYTB, ND2, ATP8, ND3, ND6, and COX1 on the light strand. The circular mitomap was visualized via Chloroplot (9), showing transcription directions, gene locations, and GC content (Fig. 1).

**TABLE 1:** Mitochondrial genome content, organization, and codon information of *Glyptotendipes tokunagai*

Gene	Type	Minimum nucleotide position	Maximum nucleotide position	Length	Start codon	Stop codon	H/L strand^*a*^
trnI	tRNA	1	68	68	−	−	−
trnQ	tRNA	74	142	69	−	−	−
trnM	tRNA	164	232	69	−	−	−
ND2	CDS	233	1261	1,029	ATT	TAA	−
trnW	tRNA	1260	1327	68	−	−	−
trnC	tRNA	1329	1398	70	−	−	+
trnY	tRNA	1468	1534	67	−	−	+
COX1	CDS	1548	3083	1,536	TTG	TAA	−
trnL	tRNA	3096	3161	66	−	−	−
COX2	CDS	3209	3892	684	ATG	TAA	−
trnK	tRNA	3903	3974	72	−	−	−
trnD	tRNA	3984	4041	58	−	−	−
ATP8	CDS	4047	4220	174	ATT	TAA	−
ATP6	CDS	4214	4891	678	ATG	TAA	−
COX3	CDS	4931	5719	789	ATG	TAA	−
trnG	tRNA	5736	5801	66	−	−	−
ND3	CDS	5802	6155	354	ATT	TAA	−
trnA	tRNA	6156	6222	67	−	−	−
trnR	tRNA	6223	6289	67	−	−	−
trnN	tRNA	6337	6406	70	−	−	−
trnS	tRNA	6407	6473	67	−	−	−
trnE	tRNA	6482	6550	69	−	−	−
trnF	tRNA	6585	6652	68	−	−	+
ND5	CDS	6675	8411	1,737	GTG	TAA	+
trnH	tRNA	8412	8478	67	−	−	+
ND4	CDS	8497	9834	1,338	ATG	TAA	+
ND4L	CDS	9828	10121	294	ATG	TAA	+
trnT	tRNA	10126	10190	65	−	−	−
trnP	tRNA	10191	10257	67	−	−	+
ND6	CDS	10265	10798	534	ATT	TAA	−
CYTB	CDS	10801	11937	1,137	ATG	TAA	−
trnS	tRNA	11983	12050	68	−	−	−
ND1	CDS	12093	13031	939	ATT	TAA	+
trnL	tRNA	13035	13101	67	−	−	+
l-rRNA	rRNA	13103	14447	1,345	−	−	+
trnV	tRNA	14480	14551	72	−	−	+
s-rRNA	rRNA	14552	15241	690	−	−	+

^*a*^ +, heavy (H) strand; −, light (L) strand.

## Data Availability

The mitochondrial genome of *Glyptotendipes tokunagai* has been completely sequenced and deposited in GenBank with accession number PV781185. The reads deposited are for the whole organism. For this genome, the corresponding BioProject, SRA, and BioSample accessions are PRJNA1291952, SRR34564647, and SAMN49982289, respectively.
